# Supplementary material for: The CXCL8/MAPK/hnRNP-K axis enables susceptibility to infection by EV-D68, rhinovirus, and influenza virus in vitro
Source: Nat Commun. 2025 Feb 17;16:1715. doi: 10.1038/s41467-025-57094-0 (PMC11832783; doi:10.1038/s41467-025-57094-0)
Supplement: Supplementary file 1 — Supplementary Information [file 41467_2025_57094_MOESM1_ESM.pdf]

Supplementary Information

The CXCL8/MAPK/hnRNP-K axis enables susceptibility to infection by EV-D68, rhinovirus, and influenza virus in vitro

Yang et. al

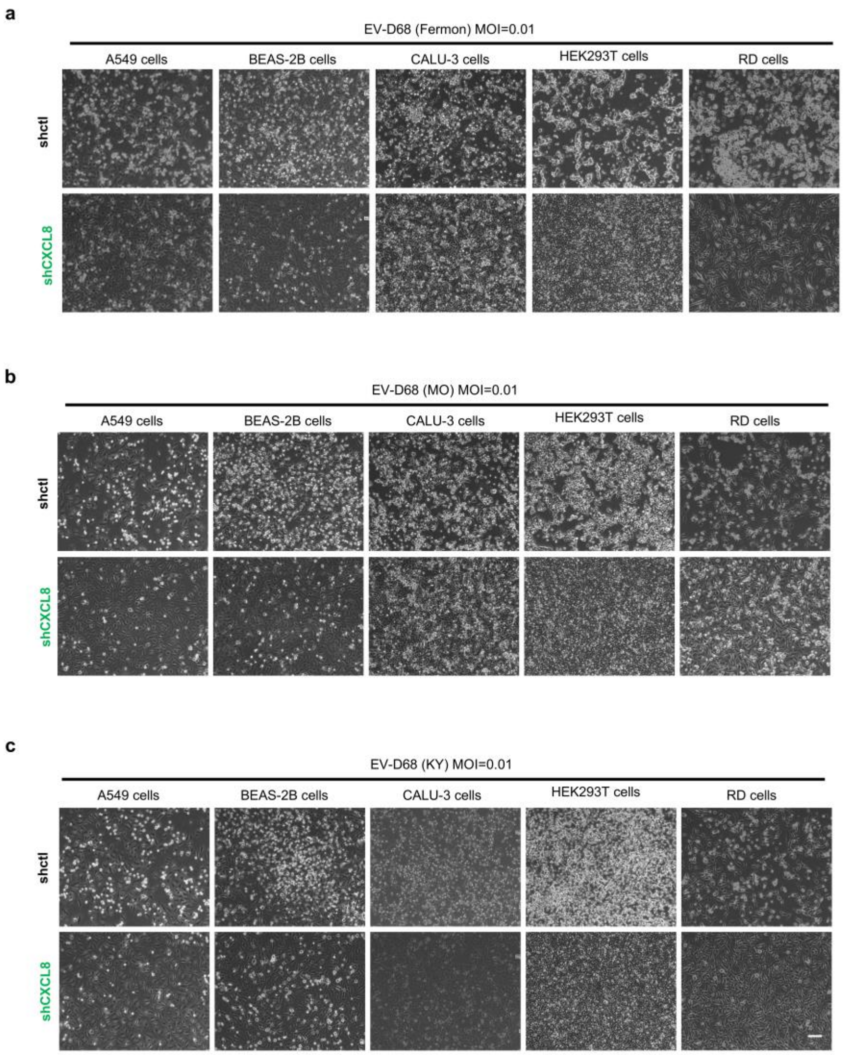

Supplementary Figure 1. CXCL8 is required for EV-D68-induced cytopathic effects. The EV-D68 prototype strain Fermon (a) and the circulating strains MO (b) and KY (c) were used to infect cells with a physiological CXCL8 level or CXCL8 knocked-down. Cytopathic effects were observed at 48 hours post-infection. Scale bar, 100 μm. Representative of three biologically independent experiments.

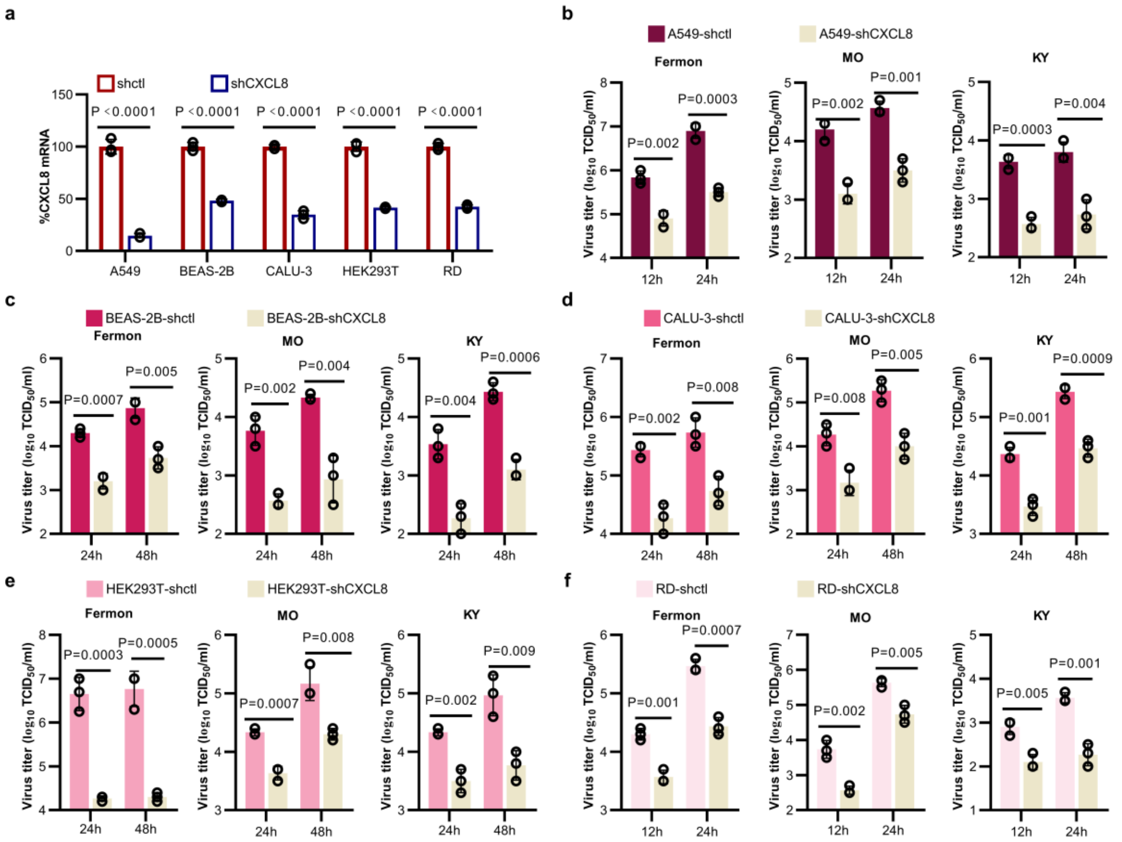

Supplementary Figure 2. CXCL8 silencing reduces progeny virion production. (a) The knockdown efficiency of CXCL8 was determined via qRT–PCR. (b-f) CXCL8 silencing decreased the virus titre in A549 cells (b), BEAS-2B cells (c), CALU-3 cells (d), HEK293T cells (e) and RD cells (f). The indicated cells were infected with the Fermon, MO, or KY strain at an MOI of 0.01. The viral titre in the supernatant was measured at different time points. *N*=3 (a-f) biological replicates. Data are represented as mean±SD. Two-tailed *t*-test (a-f) was used to assess statistical significance. Source data are provided as a Source Data file.

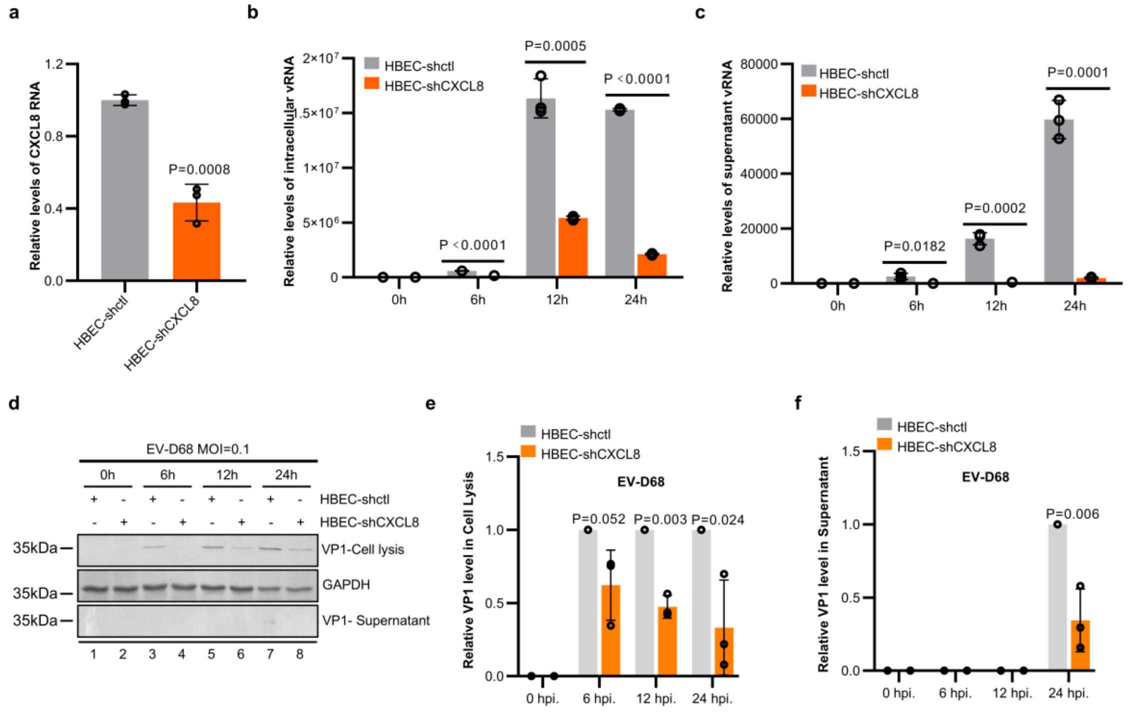

Supplementary Figure 3. CXCL8 is essential for EV-D68 replication in primary HBECs. (a) The knockdown efficiency of CXCL8 in HEBCs was determined via qRT–PCR. (b-c) Silencing of CXCL8 inhibited EV-D68 replication in HBECs. HBEC-shctl and HBEC-shCXCL8 cells were infected with EV-D68 at an MOI of 0.1, and samples from the cells and supernatants were collected 6, 12, and 24 hours post-infection. qRT–PCR was performed to evaluate the relative levels of viral mRNA in the cells (b) and supernatants (c). (d) Immunoblotting analysis was conducted to measure the abundance of EV-D68 VP1 in both the cells and the supernatant. (e-f) Quantitative analysis of relative VP1 protein levels in (d). *N*=3 (a-c, e-f) biological replicates. Representative of three biologically independent experiments (d). Data are represented as mean±SD. Two-tailed *t*-test (a-c, e-f) was used to assess statistical significance. Source data are provided as a Source Data file.

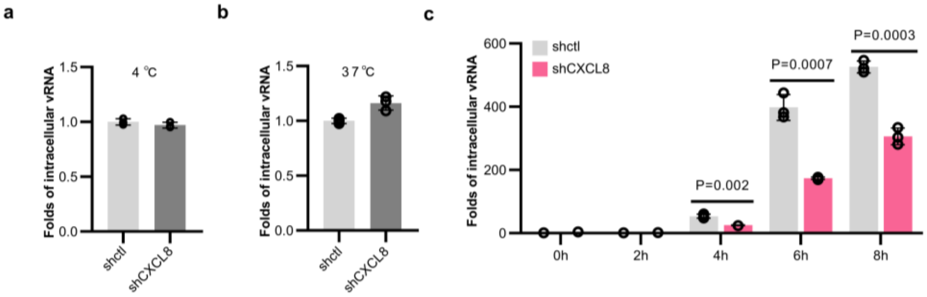

Supplementary Figure 4. The replication of EV-D68 is facilitated by CXCL8 following viral entry. (a-b) The cellular attachment and entry of EV-D68 are not affected by CXCL8. A549-shctl and A549-shCXCL8 cells were infected with EV-D68 (MOI=1) at 4°C (a) or 37°C (b). Cell samples were collected 2 hours post-infection, and relative viral mRNA levels were determined via qRT–PCR. (c) CXCL8 facilitates the early stages of EV-D68 RNA replication. A549-shctl and A549-shCXCL8 cells were infected with EV-D68 (MOI=10), and cell samples were harvested 0, 2, 4, 6 or 8 hours post-infection. qRT–PCR analysis was performed to determine relative intracellular viral mRNA levels. *N*=3 (a-c) biological replicates. Data are represented as mean±SD. Two-tailed *t*-test (c) was used to assess statistical significance. Source data are provided as a Source Data file.

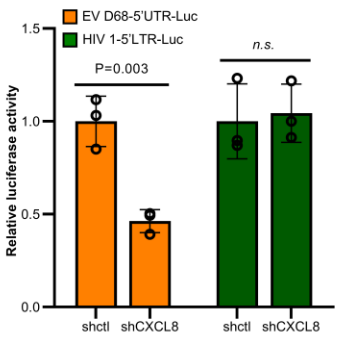

Supplementary Figure 5. Silencing CXCL8 selectively inhibits the activity of the EV-D68 5' UTR but does not influence the activity of the HIV-1 5'LTR. HEK293T-shctl and HEK293T-shCXCL8 cells were transfected with pHIV-1 5'LTR-Luc or pol I-EV68 5'UTR-Luc and Renilla luciferase. The relative fluorescence intensity was determined 24 hours after transfection. *N*=3 biological replicates. Data are represented as mean±SD. Two-tailed *t*-test was used to assess statistical significance. Source data are provided as a Source Data file.

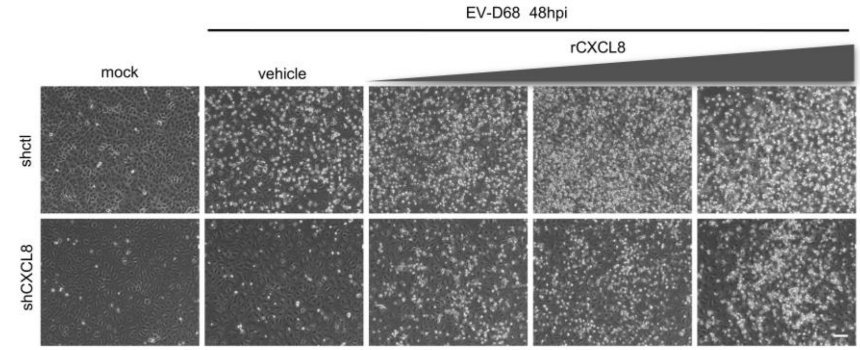

Supplementary Figure 6. rCXCL8 restores EV-D68 replication. A549-shctl and A549-shCXCL8 cells were treated with recombinant CXCL8 (rCXCL8) before EV-D68 infection. Cytopathic effects were observed at 48 hours post-infection. Scale bar, 100 μm. Representative of three biologically independent experiments.

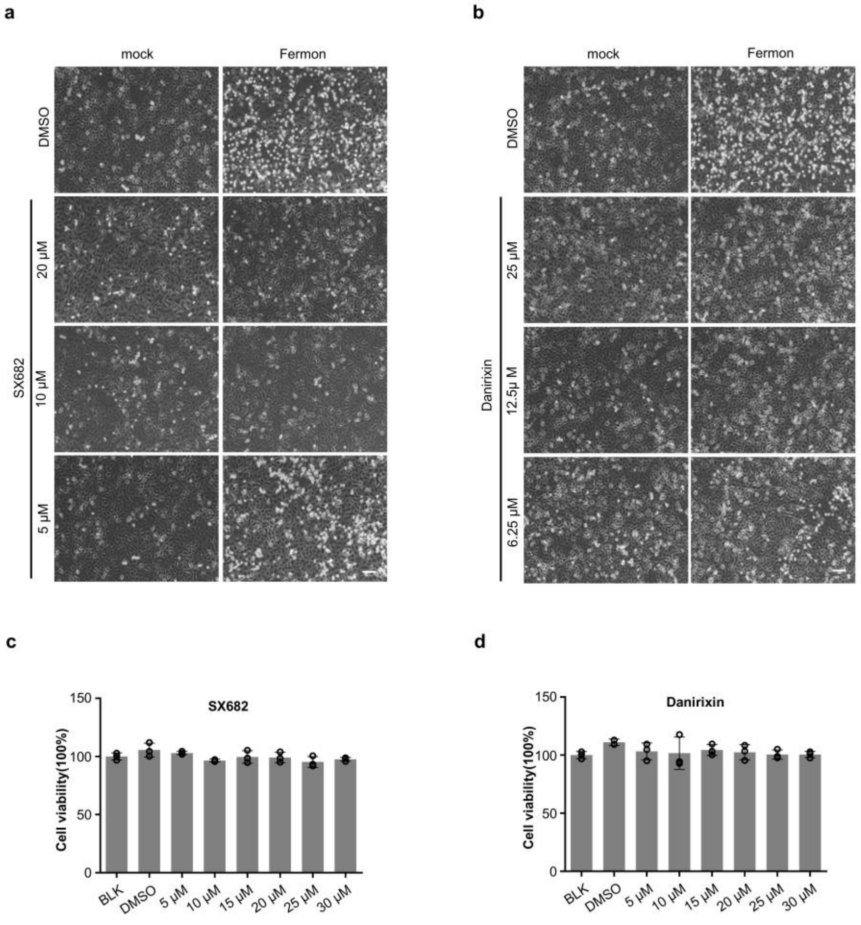

Supplementary Figure 7. CXCR1/2 antagonists suppress EV-D68 replication. (a-b) A549 cells were treated with SX682 (a) or Danirixin (b) before EV-D68 infection. Cytopathic effects were evaluated, and cytotoxicity assays were performed. (c-d) Cytotoxicity assays with SX682 (c) and Danirixin (d). *N*=3 biologically independent samples are shown in (c-d). Representative of 3 biologically independent experiments (a-b). Scale bar in (a and b) is 100 μm. Source data are provided as a Source Data file.

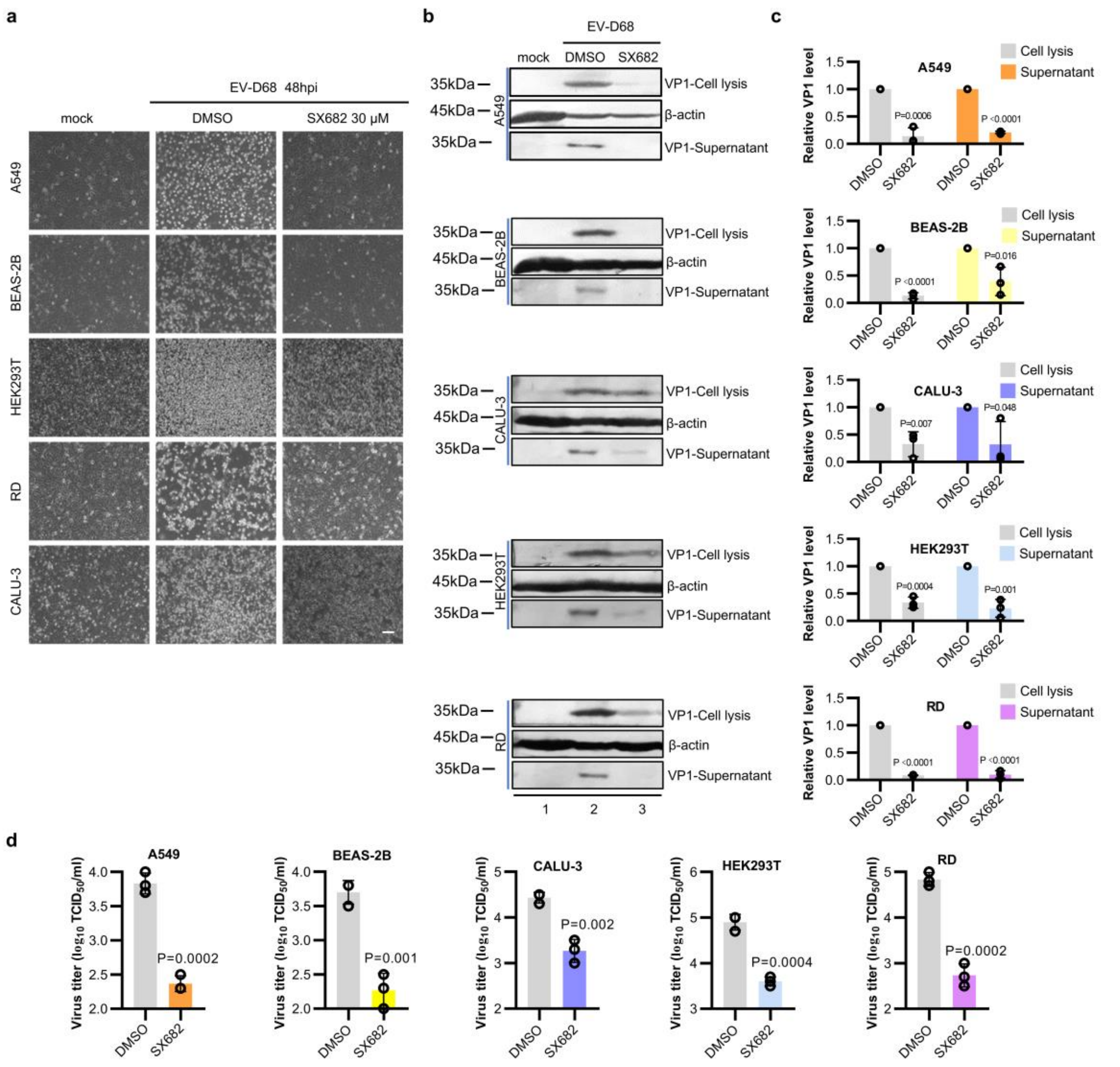

Supplementary Figure 8. SX682 suppresses EV-D68 infection across various cell types. (a-b, d) A549, BEAS-2B, 293T, CALU-3 or RD cells were treated with either DMSO or 30 μM SX682 for 30 minutes and subsequently infected with EV-D68 (MOI=0.01) or mock-infected. At 48 hours post-infection, cytopathic effects were observed (a), immunoblotting was performed to measure the abundances of the viral protein VP1 in both the cells and the supernatants (b), and the viral titre was measured (d). (c) Quantitative analysis of relative VP1 protein levels in (b). *N*=3 (c-d) biological replicates. Representative of three biologically independent experiments (a-b). Data are represented as mean±SD. Two-tailed *t*-test was used to assess statistical significance in (c-d). Scale bar in (a) is 100 μm. Source data are provided as a Source Data file.

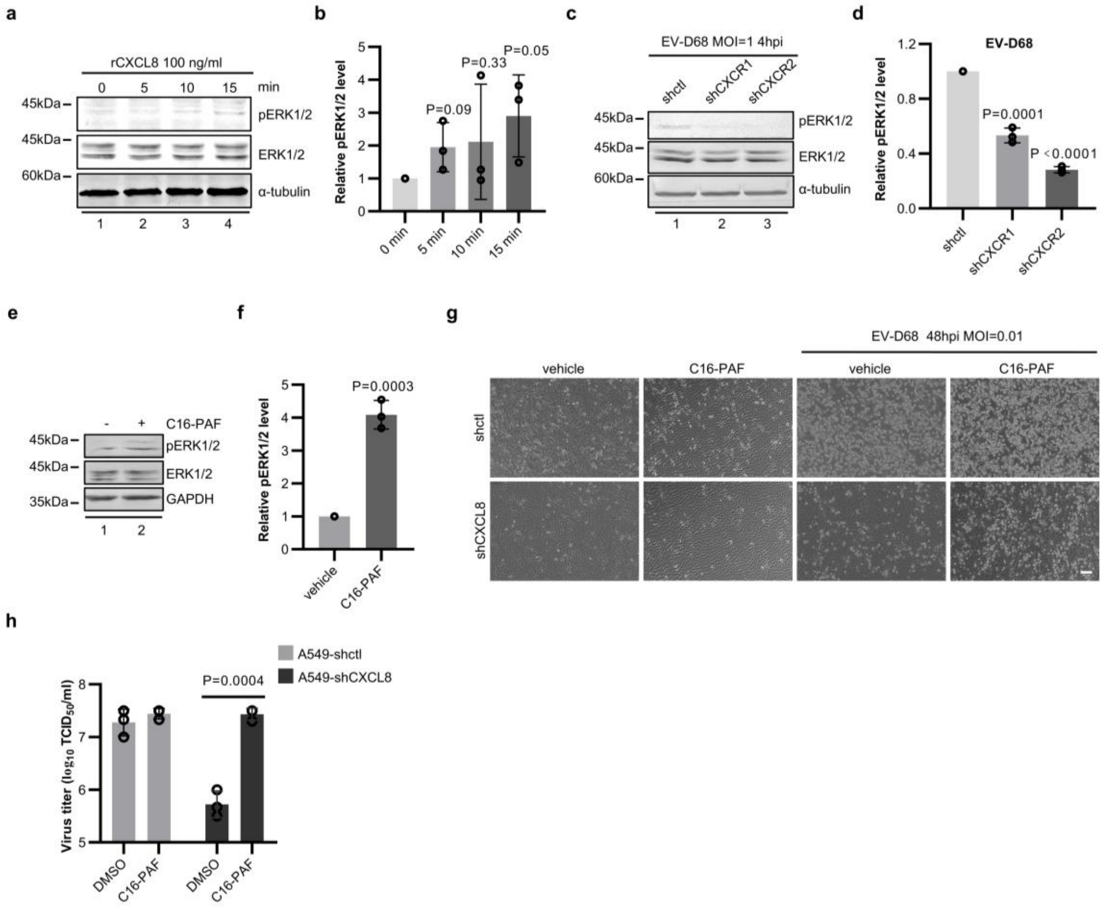

Supplementary Figure 9. The CXCL8-CXCR1/2-MAPK axis regulates EV-D68 replication. (a) rCXCL8 treatment increases ERK phosphorylation in the absence of EV-D68 infection. (c) CXCR1/2 knockdown inhibits ERK signaling activation. Samples of A549-shctl, A549-shCXCR1, and A549-shCXCR2 cells were collected 4 hours post-infection with EV-D68 (MOI=1), after which immunoblotting analysis was performed to measure the protein levels of pERK1/2 and ERK1/2. (e, g, h) C16-PAF (100 nM) facilitates the activation of the MAPK pathway and increases EV-D68 replication in CXCL8-silenced cells. (b, d, f) Quantitative analysis of relative pERK1/2 protein levels in (a, c, e). *N*=3 biologically independent samples are shown in (b, d, f, h). Representative of three biologically independent experiments (a, c, e, g). Data are represented as mean±SD. Two-tailed *t*-test was used to assess statistical significance in (b, d, f, h). Scale bar in (d) is 100 μm. Source data are provided as a Source Data file.

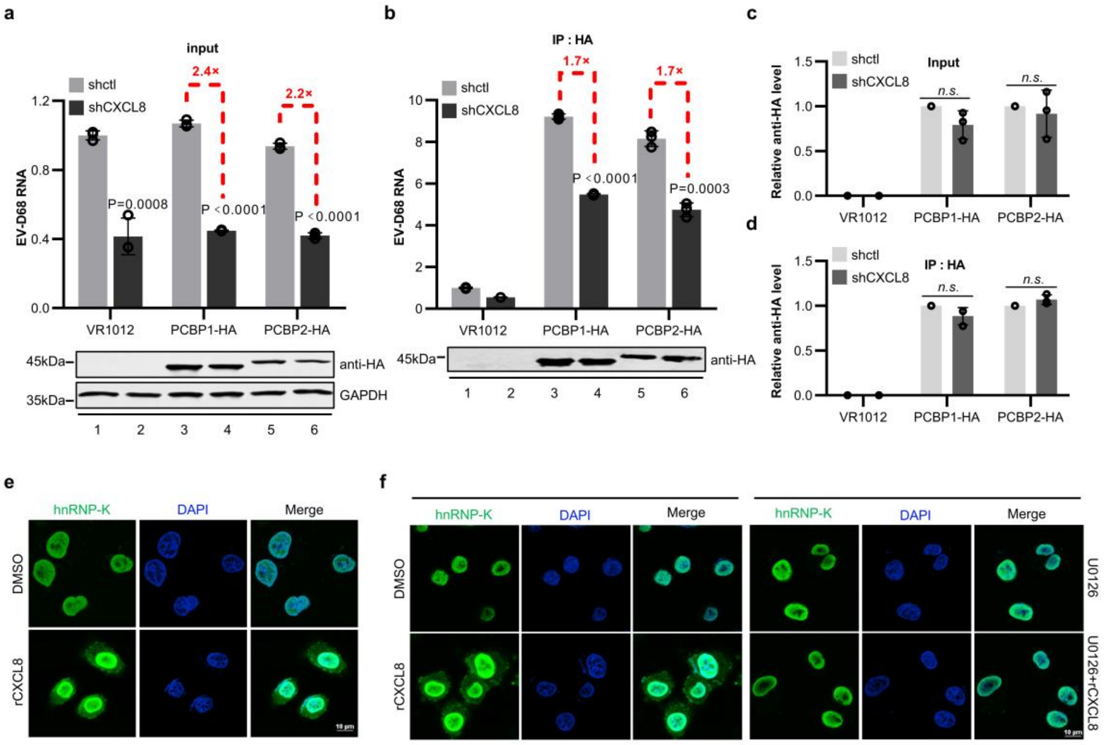

Supplementary Figure 10. The impact of CXCL8 inhibition on host factors known to bind enteroviral RNA. (a-b) The presence of CXCL8 does not influence the interaction between either PCBP1 or PCBP2 and EV-D68. (c-d) Quantitative analysis of relative protein levels in (a and b). (e) rCXCL8 treatment induces the translocation of hnRNP-K. (f) Treatment with U0126 (40 μM) inhibits the rCXCL8-mediated cytoplasmic relocalization of hnRNP-K. *N*=3 biologically independent samples are shown in (a-d). Representative of three biologically independent experiments (e, f). Data are represented as mean±SD. Two-tailed *t*-test was used to assess statistical significance in (a-d). Scale bar in (e and f) is 10 μm. Source data are provided as a Source Data file.

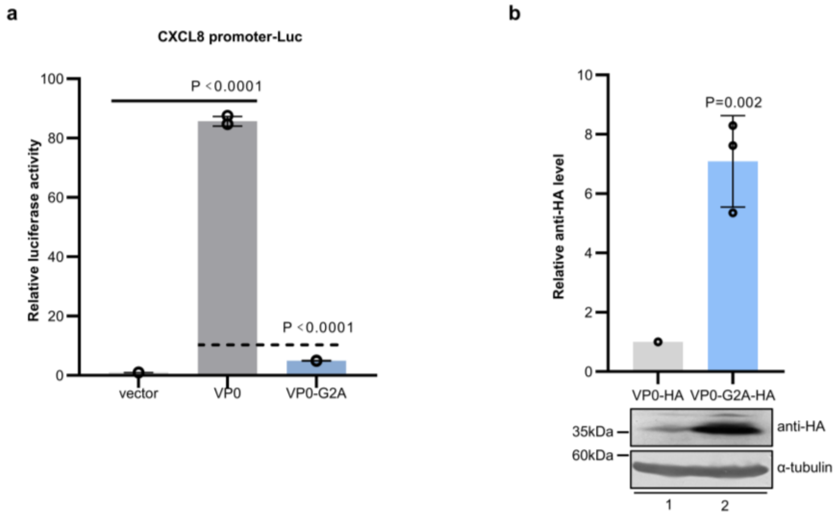

Supplementary Figure 11. Myristoylation is required for VP4-mediated CXCL8 promoter activation. (a) Cells were co-transfected with 500 ng of empty vector/VP0/VP0-G2A, 500 ng of CXCL8-promoter-Luc, or 10 ng of Renilla luciferase. Luciferase activity was measured 24 hours post-transfection. (b) Representative Western blot images depicting VP0-HA or VP0-G2A-HA expression levels in cells transfected with indicated plasmids, along with corresponding quantitative analysis of relative VP0 or VP0-G2A protein levels. *N*=3 biological replicates (a-b). Data are represented as mean±SD. Two-tailed *t*-test was used to assess statistical significance in (a-b). Source data are provided as a Source Data file.

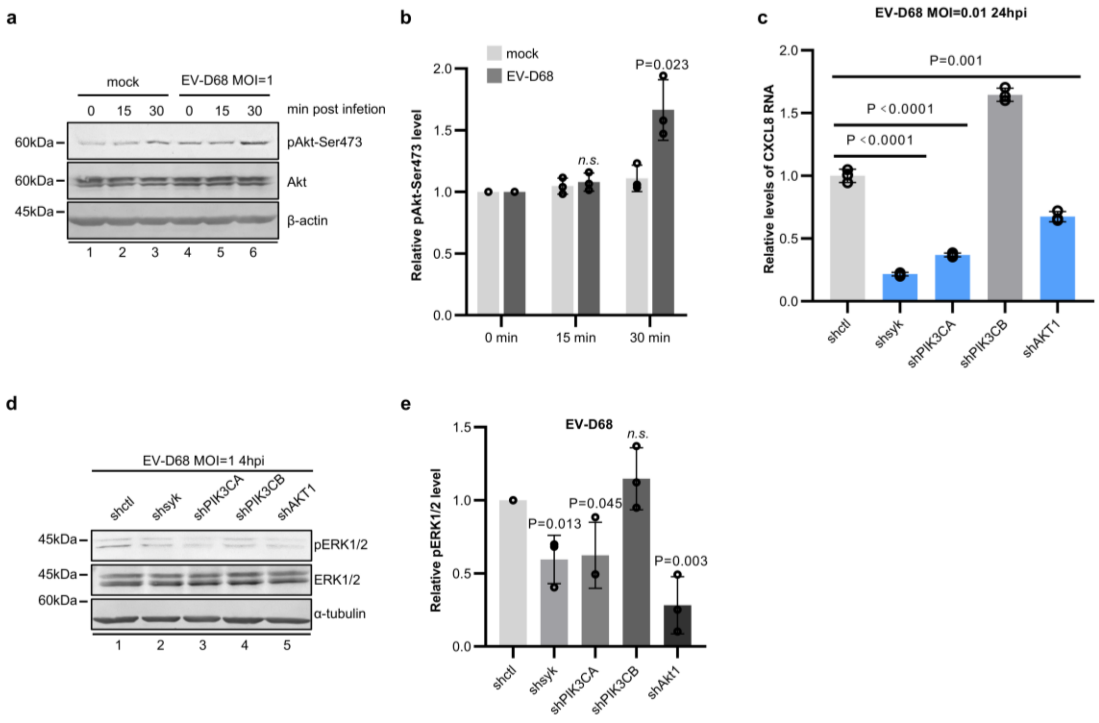

Supplementary Figure 12. EV-D68 promotes syk-PI3K-Akt signaling activation. (a) A549 cells were infected with EV-D68 at an MOI of 1, and the protein levels of Akt and pAkt-Ser473 were examined. (b) Quantitative analysis of the relative pAkt-Ser473 protein levels in (a). The value at 0 min post-infection was set as 1 for each group. (c) EV-D68 stimulates CXCL8 production through the syk-PIK3CA-AKT axis. A549-shctl, A549-shsyk, A549-shPIK3CA, A549-shPIK3CB and A549-shAKT1 cells were infected with EV-D68 at an MOI of 0.01. The relative intracellular mRNA level of CXCL8 was measured. (d) Knockdown of syk, PIK3CA or AKT1 impaired ERK pathway activation. A549-shctl, A549-shsyk, A549-shPIK3CA, A549-shPIK3CB and A549-shAKT1 cells were infected with EV-D68 at an MOI of 1. The intracellular protein levels of ERK1/2 and pERK1/2 were measured by immunoblotting. (e) Quantitative analysis of the relative pERK1/2 protein levels in (d). *N*=3 biological replicates (b-c, e). Representative of three biologically independent experiments (a, d). Data are represented as mean±SD. Two-tailed *t*-test was used to assess statistical significance (b-c, e). Source data are provided as a Source Data file.

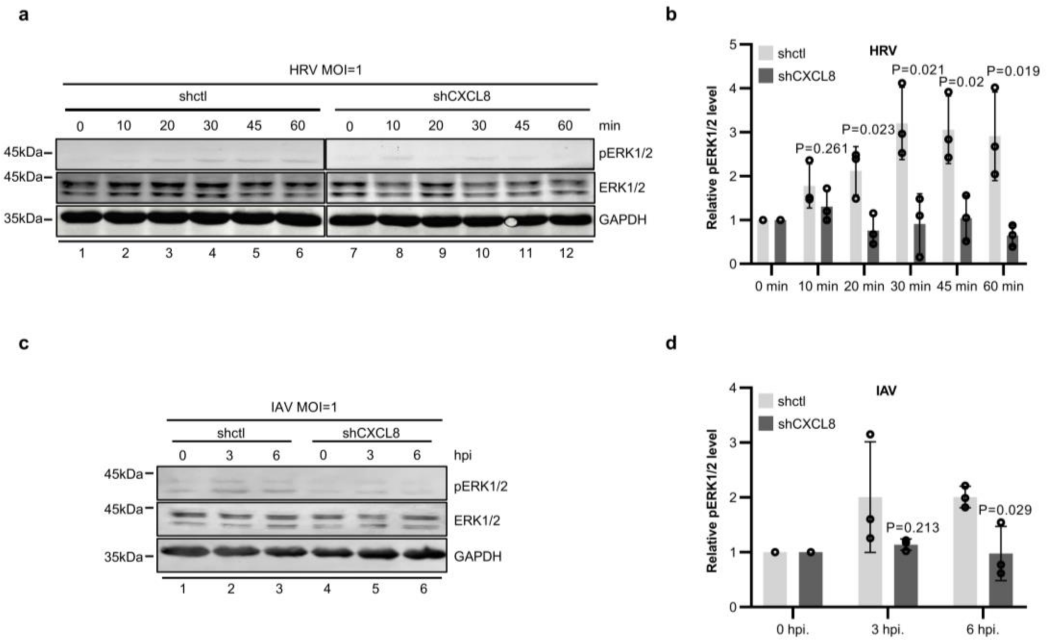

Supplementary Figure 13. Infection with HRV or IAV triggers the activation of MAPK signaling in a CXCL8-dependent manner. Representative of three biologically independent experiments (a and c). (b, d) Quantitative analysis of the relative pERK1/2 protein levels in (a and c). The value at 0 min post-infection was set as 1 for each group. *N*=3 biological replicates (b, d). Data are represented as mean±SD. Two-tailed *t*-test was used to assess statistical significance (b, d). Source data are provided as a Source Data file.

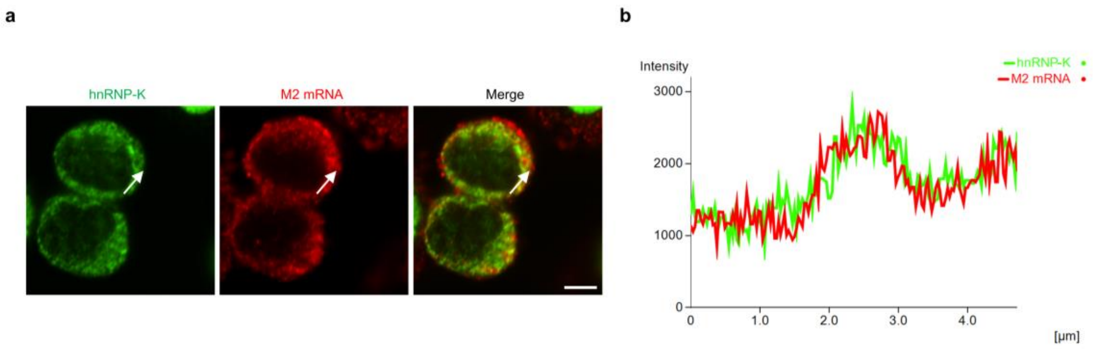

Supplementary Figure 14. Colocalization of the hnRNP-K protein and M2 mRNA in the cytoplasm. (a) A549 cells were infected with IAV at an MOI of 0.15. The subcellular localization of the hnRNP-K protein and M2 mRNA was visualized via RNA FISH and immunofluorescence staining, respectively. The scale bar represents 4.5 μm. (b) Quantitative analysis was performed to determine the fluorescence intensity of the hnRNP-K protein and M2 mRNA in the indicated region shown in Fig. S15a (white arrow). Representative of three biologically independent experiments (a, b). Source data are provided as a Source Data file.

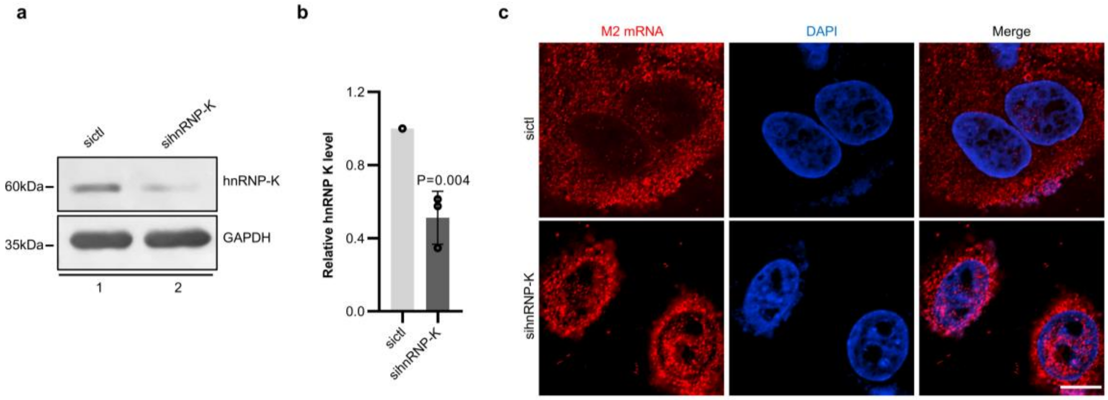

Supplementary Figure 15. Silencing hnRNP-K leads to disruption of the cytoplasmic translocation of IAV M2 mRNA. (a) Endogenous hnRNP-K was silenced by transfection with hnRNP-K-targeted siRNA. (b) Quantitative analysis of the relative hnRNP K protein levels in (a). *N*=3 biological replicates. (c) hnRNP-K-silenced A549 (sihnRNP-K) cells and control (sictl) cells were infected with IAV at an MOI of 0.15, and RNA FISH was performed to detect intracellular M2 mRNA. Representative of three biologically independent experiments (a, c). Two-tailed *t*-test was used to assess statistical significance (b). Data are represented as mean±SD. Scale bar in (c) is 5 μm. Source data are provided as a Source Data file.

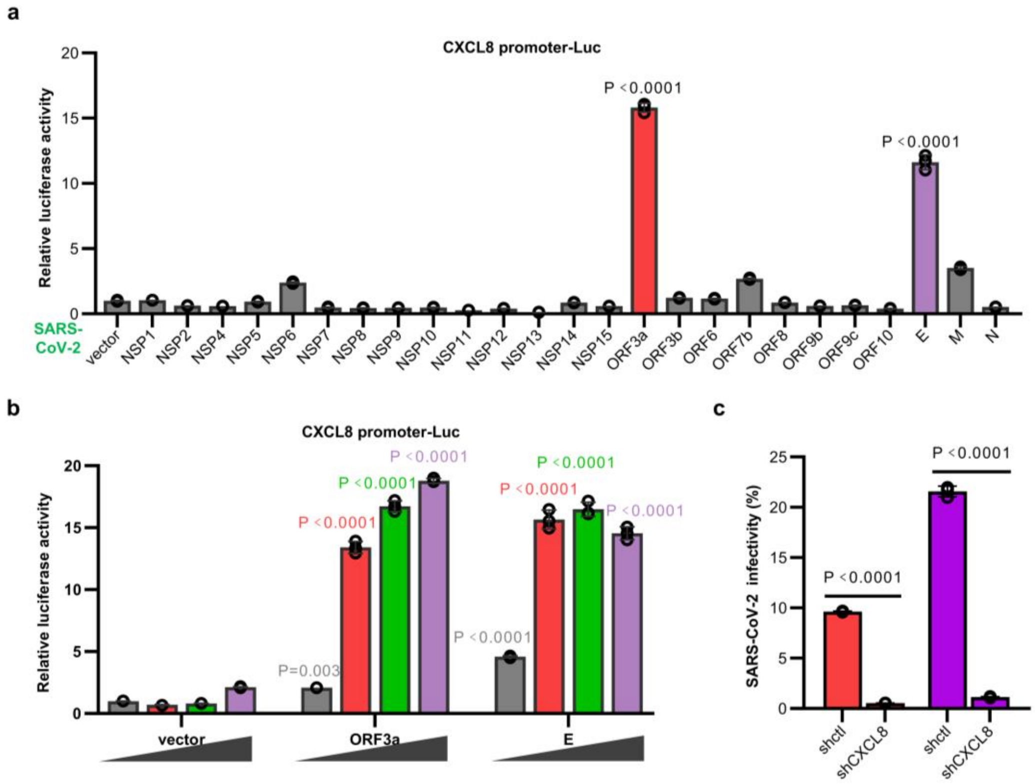

Supplementary Figure 16. CXCL8 is required for SARS-CoV-2 replication. (a-b) The viral proteins E and ORF3a of SARS-CoV-2 increase CXCL8 promoter activity in a dose-dependent manner. Cells transfected with vector were set as control groups. (c) CXCL8 expression was required for SARS-CoV-2 replication in a replication-competent SARS-CoV-2 virus-like-particle (trVLP) system. *N*=3 biologically independent samples are shown in (a-c). Data are represented as mean ± SD. Two-tailed *t*-test (a and c) or two-way ANOVA (b) was used to assess statistical significance. Source data are provided as a Source Data file.

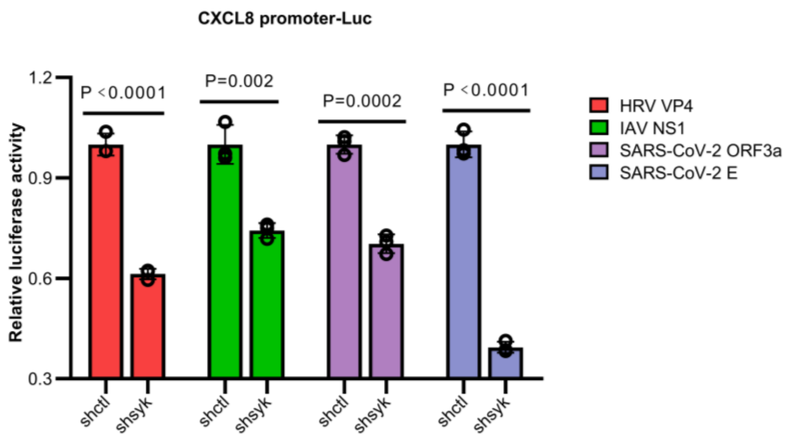

Supplementary Figure 17. Syk silencing attenuates CXCL8 activation via respiratory viral proteins. Shctl and shsyk cells were transfected with 500 ng of HRV VP4/IAV NS1/SARS-CoV-2 ORF3a/SARS-CoV-2 E, 500 ng of CXCL8-promoter-Luc and 10 ng of Renilla luciferase. Luciferase activity was measured 24 hours post-transfection (*n*=3 biological replicates). Data are represented as mean ± SD. Two-tailed *t*-test was used to assess statistical significance. Source data are provided as a Source Data file.

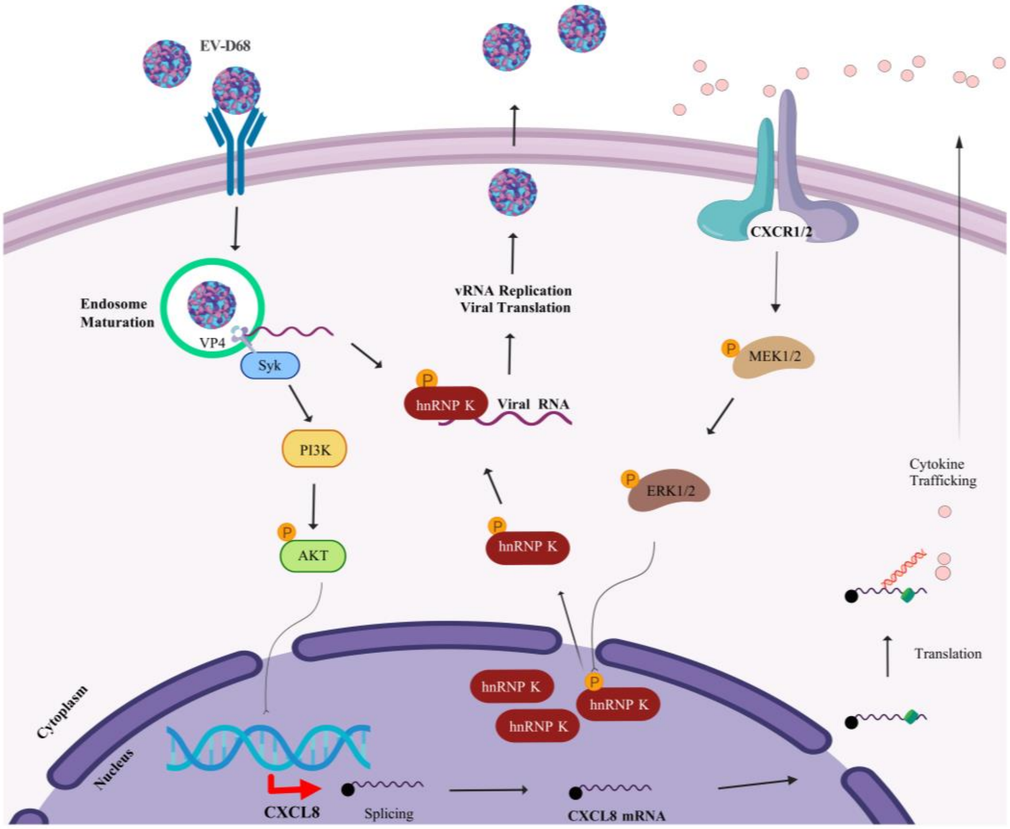

Supplementary Figure 18. Schematic representation of EV-D68 infection-induced activation of the CXCL8 signaling pathway, facilitating viral replication. This graphic was created with MedPeer (medpeer.cn). Publication License No.: 19ef10l7y4hjaidgqt1732669899.
