# Supplementary material for: The CXCL8/MAPK/hnRNP-K axis enables susceptibility to infection by EV-D68, rhinovirus, and influenza virus in vitro
Source: Nat Commun. 2025 Feb 17;16:1715. doi: 10.1038/s41467-025-57094-0 (PMC11832783; doi:10.1038/s41467-025-57094-0)
Supplement: Supplementary file 2 — Reporting Summary [file 41467_2025_57094_MOESM2_ESM.pdf]

nature portfolio

Corresponding author(s): Wei Wei

Last updated by author(s): Nov 30, 2024

Reporting Summary

Nature Portfolio wishes to improve the reproducibility of the work that we publish. This form provides structure for consistency and transparency in reporting. For further information on Nature Portfolio policies, see our Editorial Policies and the Editorial Policy Checklist.

Statistics

For all statistical analyses, confirm that the following items are present in the figure legend, table legend, main text, or Methods section.

n/a	Confirmed	
☐	☒	The exact sample size (*n*) for each experimental group/condition, given as a discrete number and unit of measurement
☐	☒	A statement on whether measurements were taken from distinct samples or whether the same sample was measured repeatedly
☐	☒	The statistical test(s) used AND whether they are one- or two-sided<br>*Only common tests should be described solely by name; describe more complex techniques in the Methods section.*
☒	☐	A description of all covariates tested
☒	☐	A description of any assumptions or corrections, such as tests of normality and adjustment for multiple comparisons
☐	☒	A full description of the statistical parameters including central tendency (e.g. means) or other basic estimates (e.g. regression coefficient) AND variation (e.g. standard deviation) or associated estimates of uncertainty (e.g. confidence intervals)
☐	☒	For null hypothesis testing, the test statistic (e.g. *F*, *t*, *r*) with confidence intervals, effect sizes, degrees of freedom and *P* value noted<br>*Give P values as exact values whenever suitable.*
☒	☐	For Bayesian analysis, information on the choice of priors and Markov chain Monte Carlo settings
☒	☐	For hierarchical and complex designs, identification of the appropriate level for tests and full reporting of outcomes
☒	☐	Estimates of effect sizes (e.g. Cohen's *d*, Pearson's *r*), indicating how they were calculated

Our web collection on statistics for biologists contains articles on many of the points above.

Software and code

Policy information about availability of computer code

Data collection	cellSens.
Data analysis	GraphPad Prism 8.0 (GraphPad Software, Boston, Massachusetts USA, www.graphpad.com) , ImageJ (https://imagej.net/software/fiji/downloads)

For manuscripts utilizing custom algorithms or software that are central to the research but not yet described in published literature, software must be made available to editors and reviewers. We strongly encourage code deposition in a community repository (e.g. GitHub). See the Nature Portfolio guidelines for submitting code & software for further information.

Data

Policy information about availability of data

All manuscripts must include a data availability statement. This statement should provide the following information, where applicable:

- Accession codes, unique identifiers, or web links for publicly available datasets
- A description of any restrictions on data availability
- For clinical datasets or third party data, please ensure that the statement adheres to our policy

The experimental data generated in this study are provided in the main text, figures, supplementary information or source data file and are available within the article. The RNA sequencing data reported in this paper have been deposited in the GEO database under accession code GSE273371 [https://www.ncbi.nlm.nih.gov/geo/query/acc.cgi?acc=GSE273371]. Source data are provided with this paper.

Research involving human participants, their data, or biological material

Policy information about studies with human participants or human data. See also policy information about sex, gender (identity/presentation), and sexual orientation and race, ethnicity and racism.

Reporting on sex and gender	not applicable
Reporting on race, ethnicity, or other socially relevant groupings	not applicable
Population characteristics	not applicable
Recruitment	not applicable
Ethics oversight	not applicable

Note that full information on the approval of the study protocol must also be provided in the manuscript.

Field-specific reporting

Please select the one below that is the best fit for your research. If you are not sure, read the appropriate sections before making your selection.

- [x] Life sciences
- [ ] Behavioural & social sciences
- [ ] Ecological, evolutionary & environmental sciences

For a reference copy of the document with all sections, see nature.com/documents/nr-reporting-summary-flat.pdf

Life sciences study design

All studies must disclose on these points even when the disclosure is negative.

Sample size	No statistical methods were used to predetermine the sample sizes.
Data exclusions	None.
Replication	All the reported experiments were reproducible. Data reproducibility was confirmed by three independent experiments.
Randomization	No method of randomization was used.
Blinding	The investigators have not been blinded during data collection and analysis.

Reporting for specific materials, systems and methods

We require information from authors about some types of materials, experimental systems and methods used in many studies. Here, indicate whether each material, system or method listed is relevant to your study. If you are not sure if a list item applies to your research, read the appropriate section before selecting a response.

Materials & experimental systems

n/a	Involved in the study	
[ ]	[x]	Antibodies
[ ]	[x]	Eukaryotic cell lines
[x]	[ ]	Palaeontology and archaeology
[x]	[ ]	Animals and other organisms
[x]	[ ]	Clinical data
[x]	[ ]	Dual use research of concern
[x]	[ ]	Plants

Methods

n/a	Involved in the study	
[x]	[ ]	ChIP-seq
[x]	[ ]	Flow cytometry
[x]	[ ]	MRI-based neuroimaging

Antibodies

Antibodies used	anti-EV-D68 VP1 antibody (Genetex, GTX132312); anti-β-actin antibody (Sigma, A3853); anti-α-tubulin antibody (GenScript, A01410); anti-glyceraldehyde-3-phosphate dehydrogenase (GAPDH) antibody (GenScript, A01622); anti-histone H3 antibody (Abcam, ab176842); anti-HA antibody (Thermo, 71-5500); anti-MEK1/2 antibody (Cell Signaling Technology, 8727); anti-pMEK1/2 antibody (Cell Signaling Technology, 9154); anti-ERK1/2 antibody (Cell Signaling Technology, 4695); anti-pERK1/2 antibody (Cell Signaling Technology, 4370); anti-hnRNP K antibody (Cell Signaling Technology, 4675); anti-PI3K-p110α antibody (Cell Signaling Technology, 4249); anti-PI3K-p110β antibody (Cell Signaling Technology, 3011); anti-Akt antibody (Cell Signaling Technology, 4691); Human IL-8/CXCL8 Antibody (Catolog #: MAB208 R&D System).
Validation	For antibodies obtained from commercial sources listed in the above section, validation was performed by the vendors.

Eukaryotic cell lines

Policy information about cell lines and Sex and Gender in Research

Cell line source(s)	Human lung adenocarcinoma A549 cells (CCL-185, ATCC), human bronchial epithelial BEAS-2B cells (CRL-9609, ATCC), human bronchial adenocarcinoma CALU-3 cells (HTB-55, ATCC), human rhabdomyosarcoma RD cells (CCL-136, ATCC), HEK293T cells (CRL-3216, ATCC), H1-HeLa cells (CRL-1958, ATCC), Madin–Darby canine kidney (MDCK) cells (GN023, Stem Cell Bank, Chinese Academy of Sciences) and Caco-2-N cells (provided by Dr Qiang Ding) . Primary HBECs, isolated from nonsmoking healthy donors and commercially available from ScienCell (Cat# 3210).
Authentication	The cell lines were authenticated by the vendors and provider. No further authentication was conducted in our lab.
Mycoplasma contamination	All cell lines were routinely tested for mycoplasma contamination. None of the cell lines used in the reported experiments tested positive.
Commonly misidentified lines (See ICLAC register)	No commonly misidentified cell lines were used.

Plants

Seed stocks	not applicable
Novel plant genotypes	not applicable
Authentication	not applicable
